# Toward Human Models of Cardiorenal Syndrome *in vitro*

**DOI:** 10.3389/fcvm.2022.889553

**Published:** 2022-05-26

**Authors:** Beatrice Gabbin, Viviana Meraviglia, Christine L. Mummery, Ton J. Rabelink, Berend J. van Meer, Cathelijne W. van den Berg, Milena Bellin

**Affiliations:** ^1^Department of Anatomy and Embryology, Leiden University Medical Center, Leiden, Netherlands; ^2^Department of Applied Stem Cell Technologies, University of Twente, Enschede, Netherlands; ^3^Department of Internal Medicine-Nephrology, Leiden University Medical Center, Leiden, Netherlands; ^4^Einthoven Laboratory of Vascular and Regenerative Medicine, Leiden University Medical Center, Leiden, Netherlands; ^5^Department of Biology, University of Padua, Padua, Italy; ^6^Veneto Institute of Molecular Medicine, Padua, Italy

**Keywords:** cardiorenal syndrome, disease modeling, hiPSCs, organ-on-chip, tissue-specific organoids

## Abstract

Heart and kidney diseases cause high morbidity and mortality. Heart and kidneys have vital functions in the human body and, interestingly, reciprocally influence each other’s behavior: pathological changes in one organ can damage the other. Cardiorenal syndrome (CRS) is a group of disorders in which there is combined dysfunction of both heart and kidney, but its underlying biological mechanisms are not fully understood. This is because complex, multifactorial, and dynamic mechanisms are likely involved. Effective treatments are currently unavailable, but this may be resolved if more was known about how the disease develops and progresses. To date, CRS has actually only been modeled in mice and rats *in vivo*. Even though these models can capture cardiorenal interaction, they are difficult to manipulate and control. Moreover, interspecies differences may limit extrapolation to patients. The questions we address here are what would it take to model CRS *in vitro* and how far are we? There are already multiple independent *in vitro* (human) models of heart and kidney, but none have so far captured their dynamic organ-organ crosstalk. Advanced *in vitro* human models can provide an insight in disease mechanisms and offer a platform for therapy development. CRS represents an exemplary disease illustrating the need to develop more complex models to study organ-organ interaction in-a-dish. Human induced pluripotent stem cells in combination with microfluidic chips are one powerful tool with potential to recapitulate the characteristics of CRS *in vitro*. In this review, we provide an overview of the existing *in vivo* and *in vitro* models to study CRS, their limitations and new perspectives on how heart-kidney physiological and pathological interaction could be investigated *in vitro* for future applications.

## Introduction

Heart and kidney are highly interdependent both in health and disease. They are essential for regulating cardiovascular (CV) homeostasis, maintaining hemodynamic stability and controlling fluid and nutrient perfusion of organs in the whole body (1). Heart and kidney communicate reciprocally through a variety of pathways and paracrine signaling (2). Neurohormonal activity of the atrial natriuretic peptides (ANP), renin-angiotensin-aldosterone system (RAAS), and sympathetic nervous system (SNS) finely tune their dialogue. Dysfunction or disease of the heart may initiate disease of the kidney and *vice versa* (3).

“Cardiorenal syndrome” (CRS) is an umbrella term used to describe the dysfunction between the two organs (2, 4) and it includes disorders where acute or chronic dysfunction in one organ may induce acute or chronic dysfunction of the other (5). A five-part classification system for CRS was introduced in 2008 and is summarized in Table 1 (2). CRS subtypes can be distinguished depending on the pathological conditions, time frame and nature of the concomitant cardiac and renal dysfunction.

**TABLE 1:** Classification of the clinical types of CRS.

	Type 1 Acute cardio-renal syndrome	Type 2 Chronic cardio-renal syndrome	Type 3 Acute reno-cardiac syndrome	Type 4 Chronic reno-cardiac syndrome	Type 5 Secondary CRS syndrome
Definition	Acute worsening of heart function leading to kidney injury and/or dysfunction	Chronic abnormalities in heart function leading to kidney injury or dysfunction	Acute worsening of kidney function leading to heart injury and/or dysfunction	Chronic kidney disease leading to heart injury and/or dysfunction	Systemic conditions leading to simultaneous injury and/or dysfunction of heart and kidney
Primary events	Acute heart failure Acute coronary syndrome Cardiogenic shock	Chronic heart disease (e.g., LV remodeling and dysfunctions or cardiomyopathy)	Acute kidney injury	Chronic kidney disease	Systemic disease (e.g., sepsis, amyloidosis)
Secondary events	Acute kidney injury	Chronic kidney disease	Acute heart failure Acute coronary syndrome Arrhythmias Shock	Chronic heart disease Acute heart failure Acute coronary syndrome	Acute heart failure Acute coronary syndrome Acute kidney injury Chronic heart disease Chronic kidney disease
Cardiac biomarkers	Troponin, CK-MB, BNP, NT-proBNP, MPO, IMA	BNP, NT-proBNP, C-reactive protein	BNP, NT-proBNP	BNP, NT-proBNP, C-reactive protein	C-reactive protein, procalcitonin, BNP
Renal biomarkers	Serum cystatin C, creatinine, NGAL, Urinary KIM-1, IL-18, NGAL, NAG	Serum creatinine, cystatin C, urea, uric acid, C-reactive protein, decreased GFR	Serum creatinine, cystatin C, NGAL, Urinary KIM-1, IL-18, NGAL, NAG	Serum creatinine, cystatin C, urea, uric acid, decreased GFR	Creatinine, NGAL, IL-18, KIM-1, NAG

*The table summarizes the different types of CRS, underlying the primary and secondary events occurring for each subtype. Moreover, the clinically relevant cardiac and renal biomarkers are listed. Adapted from Ronco et al. (5).*

However, the practicality and clinical applicability of this classification system has been recently questioned, as pathological cascades involving heart, kidney, and neurohormonal systems are already initiated by the time clinical manifestations are detected. Hence, it is almost impossible to define which factor was the initiator and which was its consequence. Another classification system was proposed where CRS is categorized on the response to various treatment modalities (6). Although this classification system may be of more practical value in clinical settings, this review is structured around the five-part classification system because *in vitro* one can control and investigate the role of the initiator and its consequence.

Cardiorenal syndrome causes high morbidity and mortality, yet the underlying biological mechanisms are not fully understood because complex, multifactorial, and dynamic mechanisms are involved. So far, the disease has only been modeled *in vivo*. Although animal models can capture the interaction between the organs, they remain difficult to manipulate and control. Moreover, interspecies differences in genetics, anatomy, vascular neural conduction may limit extrapolation to the human condition. Heart and kidney have each been modeled separately *in vitro* but models so far have had insufficient complexity to capture the dynamic interactions between the organs. For this reason, CRS is clearly a condition that would benefit from increased complexity of *in vitro* models that reflect organ-organ interaction “in-a-dish.”

In this review, we describe the state of the art regarding *in vivo* and *in vitro* models available to recapitulate CRS and, more generally, models of either of the two organs that demonstrate hallmarks of CRS such as oxidative stress, inflammation, and fibrosis. Importantly, we discuss new perspectives on how CRS can be investigated *in vitro* using these state-of-the-art organ models.

## Mechanisms and Hallmarks of Cardiorenal Syndrome

**FIGURE 1:**
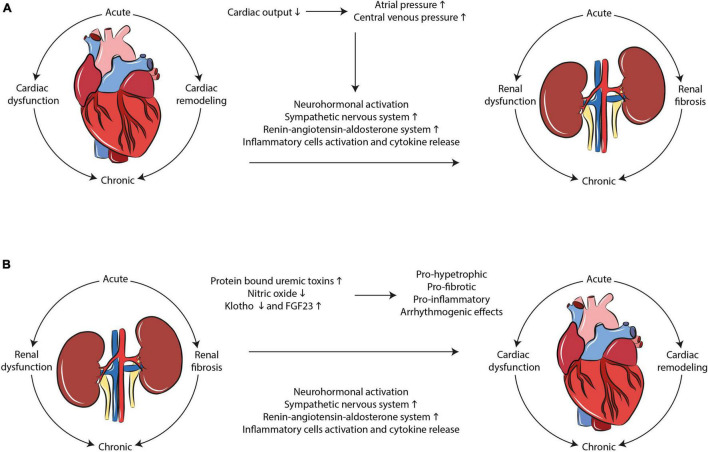
Mechanisms interplaying in CRS development. Multiple complex and dynamic mechanisms play a role in the occurrence of CRS. SNS/RAAS system activation, oxidative stress, and inflammation as well as tissue remodeling and fibrosis are the main hallmarks of the disease in both **(A)** cardio-renal and **(B)** reno-cardiac types of CRS.

Cardiorenal syndrome develops through hemodynamic and non-hemodynamic mechanisms. Hemodynamic mechanisms are defined by the interactions of blood pressure, renal function, extracellular fluid volumes, and cardiac contractility. Non-hemodynamic mechanisms include cardiorenal connectors such as RAAS, SNS, nitric oxide (NO), and reactive oxygen species (ROS) (7). While hemodynamic mechanisms may explain the adverse relationship between heart and kidney in acute failure, the interpretation of the complex physiological, biochemical, and hormonal mechanisms involved in chronic CRS remains poorly understood. RAAS, SNS activation, oxidative stress and inflammation, fibrosis and tissue remodeling represent the most important mechanisms that, upon dysregulation, lead to cardiorenal damage (Figure 1).

### Sympathetic Nervous System and Renin-Angiotensin-Aldosterone System Activation

Cardiovascular homeostasis is maintained by a set of complex and finely tuned interactions between heart and kidney, where SNS and RAAS are crucial effectors. Imbalance in RAAS and SNS is tightly coupled and, collectively, they play an important role in the development of CRS (8). Kidneys of heart failure (HF) patients release large amounts of renin with consequent increased angiotensin II production, which results in efferent arteriolar constriction and increase in oncotic pressure of peritubular capillaries. Deterioration in the kidney function was associated with elevated central renal pressure, arterial underfilling, and renal venous congestion (9). High venous pressure worsens glomerular filtration rate (GFR), suggesting that persistent RAAS and SNS activation contribute to chronic kidney disease (CKD) progression (10, 11). In acute kidney injury (AKI) patients, interactions between kidney and heart include RAAS and SNS hyperactivity. Myocardial activity is impaired by the hyperactivity of SNS with abnormal norepinephrine secretion from adrenal glands, which reduces Ca^2+^ metabolism, increases myocardial oxygen demand and myocardial cell β1-adrenergic mediated apoptosis and stimulates α1 receptors (10). RAAS activation was detected in the plasma of acute HF patients as increased plasma renin-activity and aldosterone levels. These increases were associated with greater renal failure (12). Clinical studies have also shown that SNS overactivity affects kidney function through adrenergic receptors (13). Overall, RAAS and SNS are key cardiorenal connectors, as they are induced by the bidirectional failure of heart and kidney (7).

### Oxidative Stress and Inflammation

Other cardiorenal mediators are activated in conjunction with RAAS and SNS. In patients affected by CRS, angiotensin II-induced ROS production by nicotinamide adenine dinucleotide phosphate (NADPH) oxidase causes inflammation. Chronic inflammation is a hallmark of both heart and kidney failure. Sera from CRS patients were characterized by high levels of proinflammatory cytokines and proapoptotic factors (14).

The balance between NO and ROS and inflammation is complex. NO and ROS are both involved, in opposing ways, in renal sodium handling, systemic and renal hemodynamics (7). They are also essential for the regulation of cardiac function (15). Heart and renal failure are associated with decreased NO bioavailability and oxidative stress. Oxidative stress results from the body’s ability to metabolize ROS, or when antioxidant defense mechanisms are depleted. The inactivation of the relaxing, endothelium-derived factor NO is an important effect of ROS. Anion O_2_^–^ reacts with NO and inactivates its beneficial effect by forming peroxynitrite, which oxidizes lipids, DNA and proteins (16).

Oxidative stress is a hallmark of CRS, and patients displayed increased circulating ROS and reactive nitrogen species, together with augmented expression of interleukin-6 (IL-6). Patients had increased levels of NADPH oxidase and myeloperoxidase—an enzyme catalyzing the formation of ROS—with upregulation of proinflammatory mediators (17). In AKI, circulating levels of inflammatory cytokines like TNF-α, IL-1, and IL-6 were increased after renal ischemia and, together with other cytokines and interferon-α, have direct cardio-depressant effects (18). The decreased mitochondrial oxidative metabolism in the heart is compensated by an increase in glucose uptake and glycolysis, with consequent decrease in myocardial adenosine triphosphate (ATP). Cytokines and the transforming growth factor-β (TGF-β) characterize the pro-inflammatory and pro-fibrotic state and are activated by ROS. In the kidneys, TGF-β1-induced cellular ROS is caused by NADPH oxidases and mitochondrial metabolism. The oxidative-dependent activation of transcription factors such as NF-κB and c-Jun leads to the upregulation of renal genes contributing to interstitial fibrosis and inflammation, like *phospholipase A2*, *MCP-1*, *CSF-1*, and *COX-2* (19).

### Fibrosis and Tissue Remodeling

Fibrosis is a typical tissue-remodeling process following stress and injury. It is the result of several phenomena including the epithelial-to-mesenchymal transition (EMT), fibroblast activation to produce extracellular matrix (ECM), and the recruitment of inflammatory cells with cellular regeneration at the site of damage. EMT in particular is one of the major causes of renal fibrosis: tubular epithelial cells lose epithelial properties and acquire myofibroblast characteristics by producing excessive deposition of ECM (20). Increased levels of aldosterone may cause TGF-β upregulation and increased secretion of fibronectin, causing glomerular fibrosis (21). Yet, studies suggested that renal epithelial cells undergo a partial EMT, contributing in a paracrine manner to fibrogenesis and inflammation (22).

In AKI patients, cardiac injury is characterized by myocyte apoptosis and neutrophil infiltration. The upregulation of beta-galactoside-binding lectin galectin-3 (Gal-3) expression in renal ischemia links AKI to cardiac fibrosis (10). Gal-3 is secreted by activated macrophages and is a marker of hypertrophic hearts developing HF. As in patients with chronic ischemic heart and hypertension, CKD patients develop cardiac fibrosis in the endocardium and epicardium. Uremic toxins like indoxyl sulfate and p-cresol contribute to cardiac fibrosis (10). When compared to a control population, CKD patients had a 300-fold higher concentration of indoxyl sulfate, correlated with cardiac fibrosis, TGF-β synthesis, tissue inhibitor of metalloproteinase-1 (TIMP-1) and α-1 collagen (10, 23). Upregulation of Gal-3 was also shown. In fact, Gal-3 interacts with ECM proteins like laminin, synexin, and integrins and it can bind to cardiac fibroblasts leading to increased collagen production in the myocardium (23). Cardiac remodeling and fibrosis are also associated with elevated levels of B-type natriuretic peptide (BNP) and related N-terminal pro-BNP in CKD patients when compared to cohorts with preserved renal function (24).

Cardiac fibrosis and left ventricular hypertrophy (LVH) are common features of cardiomyopathies contributing to mortality in CKD patients. Excessive cardiac fibrosis and enhanced accumulation of collagen fibrils occurs as the result of increased ECM protein synthesis, in parallel with ECM degradation regulated by matrix metalloproteinases (MMPs) and TIMPs (25). In the myocardial tissue of CKD patients, the accumulation of fibrillar collagens I and III was increased and correlated to dialysis vintage, α-Klotho deficiency and enhanced cardiac angiotensinogen expression (26). α-Klotho (hereinafter called Klotho) is a membrane protein highly expressed in kidney which functions as a co-receptor of fibroblast growth factor (FGF) receptors, activating the FGF23 signaling pathway. A decreased level of soluble Klotho in serum and urine, followed by an increase in FGF23 serves as an early marker for kidney dysfunction and predictor of CV disease. Deficiency indicates CKD progression and CV disease. Preventing Klotho decline, endogenous activation of its production or exogenous supplementation was shown to attenuate renal fibrosis and CKD progression, enhance mineral metabolism, ameliorate cardiomyopathy, and prevent vascular calcification (27).

Cardiac hypertrophy and fibrosis were linked to increased serum levels of FGF23 in CKD patients (28). FGF23 is a member of the FGF ligand family, implicated in the regulation, growth, and differentiation of cardiac myocytes. In kidney, it has paracrine functions blocking vitamin D3 synthesis. In CKD development, phosphate accumulation leads to increased FGF23 secretion, promoting LVH and cardiac remodeling (29). Enhanced myocardial expression of FGF23 and Klotho deficiency were also observed in CKD patients, which strongly correlated with LVH (30, 31). Cardiac levels of FGF23 were associated with up-regulation of FGFR24 and activation of the calcineurin NFAT signaling pathway, a mediator of cardiac remodeling and LVH (30). Moreover, changes in circulating levels and activities of phosphate regulators FGF23 and Klotho led to the development of uremic cardiomyopathy in CKD patients (32).

## Modeling Cardiorenal Syndrome Mechanisms and Hallmarks *In Vivo*

Animal models have a pivotal role in the identification of molecules related to cardiac and renal injury, helping in the elucidation of the mechanisms underlying heart-kidney interactions. Because of their availability and possibility to introduce targeted genetic mutations (e.g., knock-out, knock-in, or transgenic), the large majority of preclinical studies has been performed in either rats or mice. However, the predictive value of murine models is limited, mainly because of species-specific differences; as a result, translation of results to patients has also been limited (32). In the following section, an overview of the animal models used to study the heart and kidney interaction in disease is given and summarized in Table 2.

**TABLE 2:** *In vivo* recapitulation of CRS hallmarks.

Model	Heart to kidney dysfunction	Kidney to heart dysfunction	Combined dysfunction of heart and kidney	SNS/RAAS activation	Oxidative stress and inflammation	Fibrosis and tissue remodeling	References
Mouse	**⋅**				**⋅**		(37)
Rat	**⋅**				**⋅**	**⋅**	(39)
Mouse	**⋅**				**⋅**		(33)
Mouse	**⋅**				**⋅**	**⋅**	(35)
Mouse	**⋅**					**⋅**	(40)
Rat	**⋅**			**⋅**		**⋅**	(42)
Mouse		**⋅**			**⋅**	**⋅**	(45)
Transgenic mouse		**⋅**			**⋅**		(46)
Transgenic mouse		**⋅**			**⋅**		(47)
Rat		**⋅**			**⋅**	**⋅**	(48)
Rat		**⋅**			**⋅**	**⋅**	(49)
Mouse		**⋅**			**⋅**		(50)
Mouse		**⋅**			**⋅**		(51)
Rat		**⋅**			**⋅**	**⋅**	(52)
Mouse		**⋅**			**⋅**		(55)
Rat		**⋅**				**⋅**	(56)
Mouse		**⋅**			**⋅**	**⋅**	(57)
Mouse		**⋅**			**⋅**	**⋅**	(59)
Transgenic mouse		**⋅**			**⋅**	**⋅**	(60)
Rat		**⋅**			**⋅**	**⋅**	(62)
Mouse		**⋅**				**⋅**	(64)
Mouse		**⋅**				**⋅**	(65)
Mouse		**⋅**				**⋅**	(66)
Rat		**⋅**		**⋅**	**⋅**	**⋅**	(67)
Rat		**⋅**			**⋅**	**⋅**	(68)
Rat		**⋅**				**⋅**	(73)
Rat			**⋅**		**⋅**	**⋅**	(75)
Rat			**⋅**		**⋅**	**⋅**	(76)
Rat			**⋅**			**⋅**	(78)
Transgenic mouse			**⋅**	**⋅**			(79)
Rat			**⋅**	**⋅**	**⋅**	**⋅**	(80)
Rat			**⋅**	**⋅**			(81)

*Summarized are the in vivo models where the study of heart and/or kidney dysfunction identified hallmarks characterizing CRS (indicated with dots).*

### Animal Models Where Heart Dysfunction Affects the Kidney

To model acute cardiac dysfunction, intravenous injection of potassium chloride in mice has been widely used to induce cardiac arrest followed by cardiopulmonary resuscitation. The acute response resulted in AKI, characterized by reduced GFR and increased serum creatinine levels (33). Cardiac arrest models have provided increasing evidence of the role of an inflammatory response in acute cardiac dysfunction.

Experimental myocardial infarction (MI) is used to induce regional injury and chronic HF though the sudden occlusion of the left anterior descending coronary artery or one of its branches (34). In a mouse model of MI, the development of chronic HF was followed by left ventricular (LV) dilation and thinning of the anterior wall; reduced GFR, elevated serum creatinine associated with mild renal fibrosis and swelling of glomeruli and tubules (35). Systemic depletion of immunocompetent cells (monocytes and macrophages) attenuated renal fibrosis, suggesting their role in its development after MI. The TGF-β/Smad/NF-κB pathway might be mediating renal inflammation and fibrosis (36). A commonly shared pathogenic pathway is among the primary causes of CKD and, as fibrosis increases, the nephron gradually loses its regenerative capacity and apoptosis occurs. Hence, inflammatory processes activated upon MI may result in renal fibrosis, affecting renal function (32).

In murine models of CRS type 1, circulating levels of TNF-α and monocyte chemoattractant protein-1 (MCP-1) decreased upon treatment with exogenous apela, a secretory hormone playing an important role in embryonic CV system development. Apela inhibited apoptosis, DNA damage, inflammation, and fibrosis in renal cells of AKI mice and also inhibited the expression of adhesion molecules in renal tissue of CRS mice (37, 38). In the pericardial sac, Gal-3 leads to inflammation, fibrosis and cardiac dysfunction and its effects are possibly mediated by TGF-β/Smad signaling pathways. In Gal-3-treated rats, it enhanced macrophage and mast cell infiltration, and increased cardiac interstitial and perivascular fibrosis. Gal-3 also increased TGF-β expression and Smad3 phosphorylation (39).

Transverse aortic constriction was used to examine renal response to chronic cardiac pressure overload. A stable pressure gradient across the thoracic aorta of the mouse resulted in ventricle hypertrophy and renal inflammation. Elevated serum creatinine and tubulointerstitial fibrosis characterized the resultant CKD. This model can be considered as clinically relevant for CRS type 2, as it showed the development of CKD in a non-ischemic, hypertrophic chronic HF model (40). Renal response to chronic cardiac volume overload was instead investigated through an aortic valve regurgitation model (41), where treated rats had reduced LV ejection fraction, LV enlargement, and hypertrophy. Moreover, aortic regurgitation also caused albuminuria due to glomerular podocyte injury. The inappropriate activation of the RAAS/SNS system is possibly the mechanism responsible for the onset of albuminuria under cardiac volume overload, suggesting that HF patients may be at increased risk of renal dysfunction due to sympathetic hyperactivity (42).

### Animal Models Where Kidney Dysfunction Affects the Heart

Warm ischemia-reperfusion is characterized by a sudden decline in renal function with severe injury in the straight segment of the proximal tubules (43) and it is currently the most used AKI model. Renal ischemia can be induced by clamping the renal pedicle causing damage by hypoxia; it is often associated with compromised intrarenal circulation and oxygenation (44). The resulting renal impairment causes LV fractional shortening and dilation, cardiac hypertrophy, and apoptosis. Renal ischemia-reperfusion-induced cardiac hypertrophy in mice, showed that Toll-like receptors 2 and 4 regulate the release of histones from dying renal cells and contribute to AKI-stimulated cardiac inflammation by selectively regulating the systemic inflammatory profile and NF-κB activation (45).

Toll-like receptor 4 mutant mice and T-cell deficient mice elucidated the role of inflammation in AKI development. CD4^+^ T-cells mediated post-ischemic renal injury, which was associated with increased inflammation. While both Toll-like receptor 4 mutant mice and T-cell deficient mice were resistant to AKI, expression of proinflammatory genes such as IL-6 was increased in the kidneys of mice when subjected to cardiac arrest (46, 47).

Following ischemic renal injury, inflammatory cells may activate systemic cytokines TNF-α, IL-1, and intercellular adhesion molecule 1 (ICAM-1), that can impair cardiac function by decreasing contractility and increasing myocyte apoptosis. Mitochondrial fragmentation could lead to cardiomyocyte apoptosis and cardiac dysfunction (48, 49).

In a model of CRS type 4, morphological and functional changes in myocardial mitochondria of CKD mice were observed, particularly a decrease in oxidative phosphorylation and fatty acid metabolism. High phosphate contributed to myocardial energy metabolism dysfunction by the downregulation of peroxisome proliferator-activated receptor gamma coactivator 1α (PGC1α). Alterations were attenuated *in vivo* and *in vitro* through restoration of PGC1α expression or genetic knockdown of interferon regulatory factor 1 (50).

Metabolomic analysis following ischemic cardiac injury showed inadequate oxidative phosphorylation and oxidative stress. A shift of energy depletion and oxidative stress, reflected systemically in the plasma, was detected in the metabolomic analysis of the kidney (51).

Heart remodeling in CKD is characterized by interstitial fibrosis and capillary loss. The potential pleiotropic effects on heart remodeling of erythropoietin, used to correct renal anemia, was investigated in rats. In combination with enalapril, erythropoietin caused reduced cardiac fibrosis and microvessel disease subtotal nephrectomy (STNx) rats, presumably by decreasing myocardial oxidative stress (52).

Uremic cardiomyopathy and atrial fibrillation in CKD may be caused by inadequate renal clearance and the subsequent accumulation of protein-bound uremic toxins (53). Indoxyl sulfate and p-cresyl sulfate are the most studied protein-bound uremic toxins and they were shown to have pro-hypertrophic and pro-fibrotic effects on cardiomyocytes and cardiac fibroblasts (54–57).

Other major uremic toxins contributing to CV disease are asymmetric dimethylarginine (ADMA), advanced glycation end products (AGE), and trimethyl amine N-oxide (TMAO) that induce both heart and kidney damage (58, 59). Indeed, intrarenal administration of ADMA attenuated renal fibrosis in mouse unilateral ureteral obstruction (UUO) model, whilst knockdown of *Ddah1* and *Ddah2* increased the amount of ADMA in the kidneys (60).

The development of uremic cardiomyopathy is characterized by declined renal function linked to changes in circulating levels and activities of physiological phosphate regulators FGF23 and Klotho (61). Administration of renal toxin adenine through diet induces severe CKD and vascular calcification (62, 63). Soluble Klotho protects the myocardium from pathological stress stimuli (64). Klotho-deficient mice exhibited cardiac impairment and hypertrophy before 12 weeks of age followed by fibrosis. The extent of cardiac hypertrophy and fibrosis correlated tightly with plasma phosphate concentration and inversely with plasma Klotho concentration (65). Intramyocardial and intravenous injection of FGF23 induced cardiac hypertrophy in wild type mice, while FGF receptor blockage was shown to attenuate it in STNx rats (66).

Impaired endothelial and renal NO production causes systemic NO reduction in CKD. In rats, chronic NO depletion by the administration of an NO synthase inhibitor induced hypertension, proteinuria, glomerulosclerosis, tubulointerstitial fibrosis, systolic dysfunction, and cardiac hypertrophy. Bilateral renal denervation ameliorated these changes and this was associated with decreased RAAS activation (67). Cardiac dysfunction was ameliorated by NO supplementation (68).

Despite extensive investigation, the pathogenesis of fibrotic diseases is complex and unclear. Myofibroblasts were identified by numerous studies as the cells responsible for the progression of fibrosis. The role of endothelial-to-mesenchymal transition (EndoMT) in the pathogenesis of fibrotic disorders was reviewed some years ago (69) and an earlier review considered experimental evidence of EndoMT leading to fibrotic development in animal models of kidney fibrosis (70). EndoMT characterized experimentally induced cardiac fibrosis in mice subjected to aortic banding and rats administered with isoprotenol (71, 72). A model of renal injury followed by heart failure was established by the subcutaneous administration of isoprenaline in rats. Spironolactone showed to inhibit EndoMT *via* the A2A receptor, ameliorating cardiorenal fibrosis (73). The signals triggering fibrosis in the kidney were also reviewed (22). Myofibroblasts in animal models of renal fibrosis express α-smooth muscle actin (α-SMA) and are mainly located in the renal interstitium/glomeruli. Their loss in peritubular capillaries combined with renal interstitial fibrosis decreases the oxygen diffusion rate and evoke interstitial hypoxia during advanced stages of CKD (74).

### Animal Models of the Combined Dysfunction of Heart and Kidney

The investigation of experimental models of dual insults combining elements of chronic cardiac and renal dysfunction is key to mimicking the complex disease processes that occur in individuals with comorbid conditions. To understand the pathophysiology of concomitant organ dysfunction, a rat model of MI, followed by STNx was used. ANP, TGF-β1 and collagen type I were increased. STNx induced a significant decrease in GFR, while MI accelerated STNx-resulting renal cortical tubulointerstitial fibrosis. Altogether, decreased cardiac function as well as increasing cardiac remodeling and renal tubulointerstitial fibrosis was observed in MI/STNx rats, nicely recapitulating the features of the clinical disease manifestations (75). Another model combining chronic HF and CKD was created by performing STNx in rats with doxorubicin-induced dilated cardiomyopathy (76).

Myocardial infarction accelerated renal fibrosis in STNx rats and increased the expression of kidney injury molecule 1 (77). STNx increased cardiac hypertrophy and fibrosis, resulting in impaired diastolic function. Increased expression of hypertrophic and profibrotic marker genes as well as activated MAPK expression in the LV were associated with accelerated organ deterioration (78). MI was performed in renin and angiotensinogen double-transgenic mice to elucidate the RAAS activity in the post-MI prognosis of CKD (79). Renal impairment post-MI cardiac remodeling was mediated by excessive RAAS activation. The effect of RAAS inhibitors has also been investigated (80, 81). In particular, the use of angiotensin II type 1 receptor blockers improved LV ejection fraction and cardiac remodeling associated with macrophage infiltration, inhibition of cardiac oxidative and inflammatory pathways. Although renal function did not improve, the increase in renal fibrosis was attenuated (80).

## Emerging Models of Cardiorenal Syndrome Mechanisms and Hallmarks *In Vitro*

Because of the common principles of animal development and organ physiology, *in vivo* models have led to detailed mechanistic understanding of many human diseases. However, biological processes that are specific to humans cannot be modeled in other animals. Current models of heart and kidney have been studied separately *in vitro* but are presently often too simplistic to capture complex organ crosstalk. The following section focuses on *in vitro* models used to study heart and kidney, which are summarized in Table 3. Some limitations of *in vivo* disease modeling in animals can be potentially overcome through human *in vitro* (3D) cell culture approaches using stem cells from different organs (82). The characterization of human pluripotent stem cells (hPSCs) and the more recent organoid technology provided the opportunity to build *in vitro* models of human specialized tissue cells, including cardiac and kidney, and to study human disease in controllable settings. These technologies already revealed novel disease mechanisms and more advanced techniques are being developed to capture more complex phenotypes and better mimic *in vivo* physiology.

**TABLE 3:** *In vitro* recapitulation of CRS hallmarks.

Cell type	Heart	Kidney	SNS/RAAS activation	Oxidative stress and inflammation	Fibrosis and tissue remodeling	References
Neonatal rat cardiomyocytes	**⋅**			**⋅**	**⋅**	(83)
Human myocardial tissue homogenates	**⋅**			**⋅**		(86)
Adult rat ventricular myocytes	**⋅**		**⋅**			(87)
Neonatal rat ventricular myocytes and fibroblasts	**⋅**		**⋅**		**⋅**	(28)
Human cardiac myocytes and fibroblasts from CKD patients	**⋅**		**⋅**		**⋅**	(26)
Neonatal rat ventricular myocytes and fibroblasts	**⋅**		**⋅**		**⋅**	(65)
Neonatal rat ventricular myocytes and fibroblasts	**⋅**		**⋅**		**⋅**	(88)
Human kidney proximal tubular epithelial cells		**⋅**	**⋅**		**⋅**	(88)
Neonatal rat cardiomyocytes	**⋅**			**⋅**		(89)
Human cardiac fibroblasts	**⋅**				**⋅**	(90)
Human cardiac mesenchymal stromal cells and right ventricular endomyocardial bioptic samples from ACM patients	**⋅**				**⋅**	(91)
Human iPSC-derived cardiomyocytes and primary ventricular cardiac fibroblasts	**⋅**				**⋅**	(92)
Rat proximal tubular epithelial cell line		**⋅**		**⋅**		(93)
Rat glomerular mesangial cells		**⋅**		**⋅**		(94)
Rat kidney tubular epithelial cells		**⋅**			**⋅**	(96)
Human renal glomerular endothelial cells		**⋅**		**⋅**		(37)
Human nephrectomy-derived cells		**⋅**		**⋅**		(97)
Human renal proximal tubular epithelial cells and fibroblasts		**⋅**			**⋅**	(98)
Human renal tubular epithelial cell line		**⋅**			**⋅**	(100)

*Summarized are the in vitro models of heart and kidney where hallmarks characterizing CRS (indicated with dots) were shown.*

### Cardiorenal Syndrome Mechanisms and Hallmarks Recapitulated by *in vitro* Heart Models

As discussed, RAAS, SNS, and inflammation are the most important mechanisms whose dysregulation may lead to cardiorenal damage. RAAS stimulation induces NAPDH oxidase activation, which in turn increases ROS formation as observed in neonatal rat cardiomyocytes (83) and human myocardial tissue homogenates (83–86). Moreover, SNS hyperactivity activates RAAS, leading to angiotensin II release which has a direct effect on cellular hypertrophy and apoptosis in adult rat ventricular myocytes (87). FGF23 activated local RAAS by increasing expression of *Agt*, *Ren*, *Ace*, *Ngal* in neonatal rat cardiomyocytes and cardiac fibroblasts *in vitro*. In human cardiomyocytes isolated from CKD patients, FGF23 stimulated angiotensinogen expression (28). RAAS components angiotensin II and aldosterone induced FGF23 expression in cardiac myocytes. FGF23 enhanced collagen remodeling, expression of pro-inflammatory, pro-survival, and pro-hypertrophic genes. In cultured human cardiac fibroblasts, FGF23 also stimulated cell proliferation, migration, expression of pro-fibrotic TGF-β receptor/Smad complexes and collagen synthesis (26). The effect of protein membrane Klotho was also studied in neonatal rat ventricular cardiomyocytes and cardiac fibroblasts *in vitro*. It was observed that Klotho inhibited fibrosis induced by TGF-β1, angiotensin II, or high phosphate fibrosis, while it abolished TGF-β1 or angiotensin II-induced hypertrophy (65).

The role of Wnt signaling in heart and kidney injury was investigated in parallel *in vivo* and *in vitro* models of CRS type 2. *In vitro*, Wnt3a induced multiple components of the RAAS in both primary neonatal rat ventricular cardiomyocytes and cardiac fibroblasts. Serum from transverse aortic constriction mice activated β-catenin and triggered cell injury; moreover, TNF-α inhibited Klotho, induced β-catenin activation, and cell injury. Altogether, these results identified Wnt/β-catenin signaling as a common pathogenic mediator for heart and kidney in CRS type 2 (88).

Uremic toxin accumulation plays a major role in CRS and high serum levels of uremic toxin p-cresol are associated with CV diseases. A study performed on cultured neonatal rat cardiomyocytes showed that p-cresol induced disassembly of gap junctions, increased Ca^2+^ levels and Ca^2+^-dependent protein kinase Cα activation (89).

Fibrosis was previously described as a key feature in the pathogenesis of HF. A major impediment in the study of cardiac fibrosis is the lack of reliable and high throughput *in vitro* models. In TGF-β-treated human primary cardiac fibroblasts, an increase in labeling of detectable glycosaminoglycan chain epitopes with extracellular structures associated with collagen was detected. Newly synthesized collagen fibrils are associated with glycosaminoglycans like dermatan sulfate (90). In arrhythmogenic cardiomyopathy (ACM), the myocardial tissue is replaced with fibrotic or fibro-fatty tissue and inflammatory infiltrates in the heart. ACM patient-derived tissue exhibited higher fibrotic markers and TGF-β levels, thus suggesting that TGF-β stimulation drives pro-fibrotic differentiation of cardiac stromal cells in ACM (91). In a human cardiac fibrosis-on-a-chip model, co-cultured human cardiac fibroblasts, and human induced pluripotent stem cell (hiPSC)-derived cardiomyocytes displayed a 20% increase in α-SMA-positive cell intensity relative to control upon TGF-β exposure; the cardiac fibrosis model displayed increased collagen deposition, higher tissue stiffness, loss of contractile function, and induced BNP secretion (92).

### Cardiorenal Syndrome Mechanisms and Hallmarks Recapitulated by *in vitro* Kidney Models

In the kidneys, ROS generation increases in response to specific stimuli (e.g., angiotensin II and aldosterone) and influences several physiologic processes. The primary source of ROS in the kidney cortex and medulla are the NADPH oxidase (NOX) enzymes. Upon angiotensin II and aldosterone stimulation, ROS production increases. The function of NOX-derived ROS in the kidneys can be classified into regulation of renal blood flow, alteration of cell fate and regulation of gene expression. NOX-derived ROS can alter renal cell fate by enhancing EMT by (i) MAP kinase activation, (ii) mesangial cell apoptosis, and (iii) ERK1/2 activation promoting cellular hypertrophy; this was shown in renal tubular epithelial cells (93, 94). ROS activates pro-inflammatory and fibrotic mechanisms *via* cytokines and TGF-β signaling, which in turn induces EMT, one of the major causes of renal fibrosis. In cultured rat kidney tubular epithelial cells, TGF-β1 signaling was sufficient to induce EMT (95, 96).

As mentioned above, the effect of apela on renal function and anti-inflammatory effects in CRS type 1 mice have been investigated. Moreover, this study used human renal glomerular endothelial cells to evaluate the adhesion of monocytes *in vitro*. *In vitro*, apela inhibited the expression of TNF-α, MCP-1, and intracellular adhesion molecule-1 in renal glomerular endothelial cells induced by angiotensin II. Apela also inhibited the promotion of angiotensin II on the adhesion of THP-1 cells (37).

In a study examining the validity of *in vitro* models in nephrology, kidney-derived human cells were exposed to TGF-β and/or hypoxic conditions. The expression levels of genes related to these two signaling pathways (*TGFβ1*, *SMAD3*, *SMAD7*, *COL1A1*, *HIF1α*, *EGLN1*, *EGLN3*, *HIF1AN*, *SIAH2*) were quantified. In all *in vitro* experimental groups (hypoxia, normoxia, hypoxia + TGF-β, normoxia + TGF-β), the expression of the genes was noisy with no consistent pattern. However, in the UUO animal model counterpart, TGF-β pathway-related genes were overexpressed in the ureteral obstruction group compared with the sham controls. Results suggest *in vitro* findings should be interpreted cautiously (97). Integrins play a major role in CKD tubulointerstitial fibrosis, but the underlying mechanisms are not fully understood. The hypothesis that integrins are pro-fibrotic *via* regulation of functional interactions between human renal proximal tubular epithelial cells and renal fibroblasts was tested on an *in vitro* system consisting of a contact co-culture of the two cell types. Integrin-mediated pathways can facilitate the spontaneous accumulation of ECM during fibroblast-epithelial cell interactions (98).

Myofibroblasts are crucial mediators in kidney fibrosis through production of ECM. They prime for apoptosis, expressing higher levels of molecules such as BCL-2 family member BIM, but evade apoptosis by activating pro-survival signals such as TGF-β1 (99). A TGF-β1/Smad oligodeoxynucleotide (ODN), a synthetic short DNA containing complementary sequence for Smad transcription factor and *TGF-β1* mRNA, was designed to study the role of TGF-β1/Smad signaling pathways in EMT and EndoMT. The study investigated the anti-fibrotic effect of synthetic TGF-β1/Smad ODN in parallel in a UUO-induced kidney fibrosis model *in vivo* and TGF-β1-induced *in vitro* model using renal tubular epithelial cell line NRK-52E. TGF-β1/Smad ODN treatment suppressed inflammatory response and ECM deposition. Through the suppression of TGF-β1/Smad-dependent and independent signaling pathways, the activation of myofibroblasts was inhibited. Moreover, it was demonstrated that synthetic ODN attenuated TGF-β1-induced epithelial dedifferentiation and EndoMT program *via* blocking TGF-β1/Smad signaling (100).

## The Next Step: Developing Cardiorenal Syndrome *in vitro* Models

While *in vitro* human cell culture models allow study of human specific biology, many limitations restrict their application, especially for complex, multi-organ diseases such as CRS. For example, human cardiac arrhythmia due to mutations in cardiac ion channels and their treatment can be accurately studied in monotypic cultures of 2D hiPSC-derived cardiomyocytes (101), but they can clearly not recapitulate organ-organ interaction or systemic responses. To this end, cell culture models are continuously being improved by co-culture of multiple cell types, which may or may not be derived from patients. However, human physiology is not only defined by cell types involved but also their environment. We and others have demonstrated that cardiomyocytes cultured in 3D and in combination with their natural “cellular neighbors” behave more like adult cardiomyocytes *in vivo* compared to cardiomyocytes cultured in a monolayer (102). Not only cell-cell interaction and/or cell-secreted factors are important here but also ECM and their physical environment. For example, while normal cell culture plastic used *in vitro* would present an extreme supraphysiological load to the cardiomyocytes, 3D co-culture provides a more authentic cellular niche, which mimics the physiological load of cardiomyocytes *in vivo* more closely. In engineered heart tissues this mimicry can even recapitulate the dynamic pre- and after-load that the heart continuously experiences (103).

The importance of a physiological environment and neighbors is also true for the renal counterpart. hiPSC-derived kidney organoids develop essential nephron subunits consisting of glomeruli and tubular structures. Several protocols show the simultaneous generation of multiple cell types in a single differentiation or by combining multiple progenitor populations first (104–106). While this results in different configuration of cell types in kidney organoids, they are all characterized by a higher degree of organization with segmentation, specialization and maturation compared to their 2D equivalents. The crosstalk between all cell types in the developing kidney organoid is essential. Apart from the cellular composition, kidney organoids should ideally be functionally vascularized to reach enhanced structural maturation through flow *in vivo* or *in vitro* (107–109).

Improved engineered tissues have the potential to become more precise platforms for both disease modeling and the development of new therapeutic approaches. Cells from various sources can be collected and cultured in 2D or 3D to study disease in great detail and can be manipulated very precisely (104, 110). In addition, the environment can be engineered to support the cellular components in microphysiological systems or Organ-on-Chip (OoC) microfluidic approaches. It is such combinations of control and ability to manipulate the environment and cells that is required to increase our ability to study and understand the mechanisms underlying the multiple types of CRS.

### Control and Manipulation of the Cellular Component

*In vitro* disease models can be based on multiple cell types of different sources, depending on the study of interest. The development of methods for the generation of 3D organoids has opened the way for modeling human heart and kidney development and disease *in vitro.* Since organs typically consist of multiple specialized cell types (e.g., cardiomyocytes for the heart and podocytes in Bowman’s capsule for the kidney) in combination with supporting cells (such as endothelial cells and fibroblasts, which often are also organ-specific) and cells of the immune system, the cellular components of complex *in vitro* models should include all cell types involved in the disease response. In self-organizing organoid cultures, relevant cell types are derived during organoid development or differentiation, often with some spatial arrangement reminiscent of the native organ. Such is the case for kidney organoids and several protocols for the generation of human (pluripotent and adult) stem cell-derived kidney organoids were described (104, 105, 111). The question remains how comparable these structures are to true kidney morphology. The importance of generating progenitor populations (ureteric bud and metanephric mesenchyme) separately and assembling them at a later stage resulted in “higher order” kidney organoids that showed branching morphogenesis, recapitulating the kidney development process (106). Most recently, the addition of a third progenitor population, the organotypic stromal component, demonstrates that the protocol of kidney organoid generation can be further advanced (112). The maturation of the structures *in vitro* is still challenging. While tubular cells in organoids adequately mature to uptake fluorescent dextrans from the lumen of tubules (113, 114), full maturation to adult-like tubular structures is not achieved. Advances have been made studying glomerular functionality from human hPSCs-derived organoids, with podocytes connecting with endothelial cells in glomerular structures and also showing basic filtration capacity when transplanted *in vivo* (107, 115, 116).

The level of self-organization reached by kidney organoids is still in development for cardiac 3D models. Most of cardiac tissue models require pre-differentiated cell types of interest to be mixed, in presence or absence of an extracellular or hydrogel component. Self-organization of hiPSCs into cardioids have also been demonstrated, but this technology is still relatively new (117). We recently developed a multicellular model using (pre-differentiated) hiPSC-derived cardiomyocytes, cardiac fibroblasts, and endothelial cells to create cardiac microtissues (118). Similarly, other microtissue models were built using different cell types (119, 120). Microtissues can be considered as small “pieces” of contractile myocardium in which, since they are made with predefined cell types, the cellular ratios and components can be fully controlled and their reciprocal influence and contribution to functional properties examined. For example, ACM is a genetic disease presenting with ventricular arrhythmias and sudden cardiac death. While it seems logical that the cell types responsible for the electrical and contractile activity (i.e., cardiomyocytes) cause this disease, we recently exploited the cardiac microtissue model by replacing healthy fibroblasts with cardiac fibroblasts derived from an ACM patient. This was sufficient for an arrhythmogenic phenotype to appear, therefore confirming recent concepts where cardiac fibroblasts may play an active role in disease onset and progression, and identifying cardiac fibroblasts among the cellular “culprits” underlying the disease phenotype (102, 121). This shows that control of the cellular components is crucial in dissecting and understanding disease mechanisms. For a more accurate and physiologically complex model, the possibility to include additional cell types such as smooth muscle cells, neurons as well as an immune component can be considered.

### Controlling the Environment

Advances in technology, such as microfluidics and hydrogels, enable the generation of “instructive” environments that can drive tissue topology and formation of organ-specific structures. For kidney disease modeling, microfluidics may help mimicking the fluid environment that supports tubular cell growth and provides a porous membrane support for cell polarity (122, 123). A multi-layered microfluidic system separated by a membrane was built to stimulate renal filtration in mouse kidney medullary collecting duct cells. The same device was later used to culture human primary renal epithelial cells (124, 125). A method to induce hiPSC-derived podocytes to form human glomerular chips, which mimicked the structure and function of the glomerular capillary wall, has also been described (126). A reusable microfluidic chip was employed in human proximal tubules and glomeruli which permitted renal epithelial cells to grow under different conditions (127). A stable tubule culture system was also designed, which allowed for extended expansion and human kidney tissue analysis (128). Vascularized kidney organoids obtained from hPSCs cultured under flow on millifluidic chips showed improved maturation when compared to the static controls (109). Until now, most enhanced maturation is reached when kidney organoids are functionally vascularized upon transplantation in mice and on the chorioallantonic membrane in chickens (107, 108). The latter study showed that the soft *in vivo* niche improved growth, differentiation and even vascularization of kidney organoids. By replacing this environment with compliant hydrogels, growth and differentiation were indeed improved.

Similarly, controlled environmental cues have already benefited the generation of different cardiac models, ranging from approaches similar to the above mentioned microtissues but with addition of hydrogels, to fully engineered tissue designs and chips to support bundles with different stiffness. Techniques like photolithography, bioprinting, whole-heart decellularization, micropatterning, biowires, or casting molds scaffolds can be used to build different formats of hPSC-derived engineered heart tissues (129–136). Mechanical support, pillars and elastic strips providing mechanical load and physiologically similar conditioning are used to grow engineered heart tissues; these were shown to induce hiPSC-cardiomyocyte maturation and reveal pathological features (103, 137, 138). In addition, either external or embedded electrodes can be used to electrically stimulate the cardiac tissue and thereby control the beating frequency of the otherwise spontaneously active cells. Control of the environment, as with the cellular component, is therefore essential. For example, in the previously mentioned ACM microtissue model, the arrhythmogenic phenotype was only found at relatively high stimulation frequencies (i.e., above 2 Hz). This correlates with the clinical phenotype since in patients, the trigger for arrhythmic events is usually strenuous exercise or more generally, catecholaminergic stress. The evolution of microfluidics enabled the *in vitro* bionic study of cardiac tissue. 3D printing technology allowed micro-organ tissue chip production, permitting the integration of myocardial and vascular systems (139, 140). Interestingly, a heart-on-chip device using high-speed impedance detection was used to assess cardiac drug efficacy (141). The physiological and mechanical environment of cardiomyocytes in a heart organ platform was successfully mimicked (142). Moreover, methods to design convenient and efficient chips to generate hiPSC-derived heart tissues in controlled environments are also available (143).

### Outlook: Organ-Organ Interaction and Integration of Readouts

Complex models can provide an insight in disease mechanisms and offer a platform for therapy development. Nonetheless, they may not completely recapitulate and predict the effects of a disease in the entire organism, as cells and microorganisms are isolated from their natural environment. Importantly, these models should be as simple as possible in order not to overcomplicate experimental setups, but be complex enough to capture the (disease) mechanism of interest. Microfluidic devices and microfabrication may represent the technological implementations needed to achieve organ vascularization and organ-organ interaction, which are crucial in complex disorders involving multiple organs/tissues as CRS. Multi-OoC could redefine the way human health research is done (144) and CRS is a perfect example of the need for more precise *in vitro* human models to study organ-organ interaction. Such models connect separate organ chambers together, thus resembling the interactions between different organs (145). Moreover, they could serve as pharmacokinetic and pharmacodynamic models for monitoring the response of multiple organs to pharmaceutical compounds (146, 147).

The ability to measure physiologically relevant parameters is crucial in the development of OoCs in general. When looking at studying CRS, it will be essential to be able to detect changes in both the cardiac and renal functions as necessary, in their respective compartments. Within the cardiac field, chips already exist that integrate electrodes (for measuring the electrical field) and impedance (which is an indication of contraction). In addition, most of the chips are transparent, allowing optical analysis for example to measure calcium transients. For the kidney structures on-chip, measuring nephron functionality will be challenging. Differential clearance of albumin should ideally be achieved as well removal of uremic solutes, when combined with endothelial cells (126). Alternatively, uptake assays, analysis of genetic, proteomic, and metabolic signatures need to be evaluated, as well as structural analysis of nephron segments using (electron) microscopy. For all of these assays, it is important that sampling of the medium—ideally at different time points and even locations within the chip—is possible, as well as that the chip can be opened (or is open-top) and the tissue collected for further processing.

**FIGURE 2:**
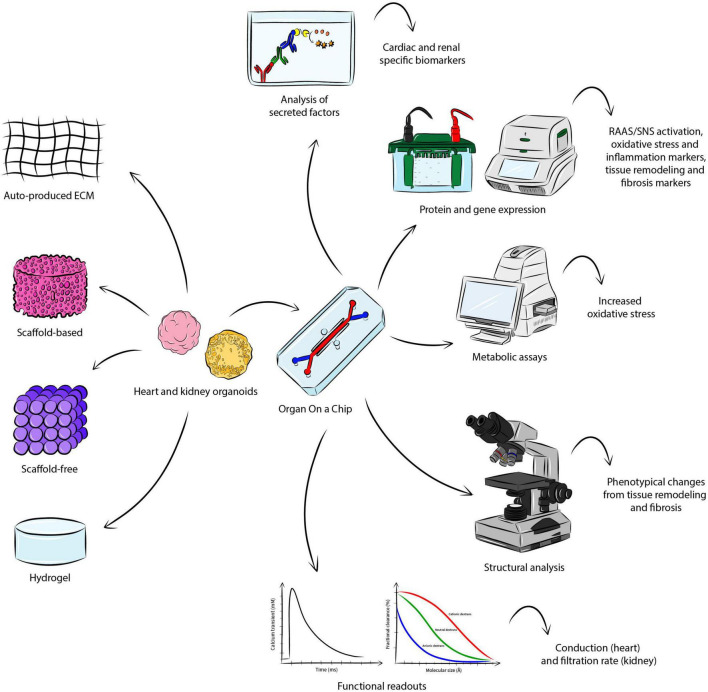
Modeling CRS *in vitro*. There are several approaches for the fabrication of heart and kidney organoids, where aggregation is either based on endogenously-produced extracellular matrix, or addition of scaffold material, or hydrogel. Coupling the engineered organoid constructs into a OoC microfluidic system may allow the establishment of CRS *in vitro*, supporting the study of the organ-organ interaction and the measurement of important parameters identifying the hallmarks of the disease. The analysis of secreted factors could detect cardio-renal biomarkers, while protein and gene expression may be investigated though Western blot and quantitative polymerase chain reaction. Metabolic assays would serve to detect a significant change in increased oxidative stress. Structural analysis would allow the visualization of phenotypical changes in the organoids. Functional analyses specific to the organoid of interest (e.g. conduction for the heart and filtration rate for the kidney) may also be conducted.

Figure 2 gives a general overview of how coupling heart and kidney organoids into a microfluidic system could support the study of CRS *in vitro*, capturing the complex interaction of the organs, allowing for the measurement of critical readouts and recapitulating the hallmarks of the disease.

## Conclusion

Cardiorenal syndrome is a broad term used to define the combined dysfunction of heart and kidney. It has recently gained attention through the realization that there is considerable reciprocal influence between the two organs that may help explaining the pathology of the disease and its variants. Although the biological mechanisms underlying the disease are complex and dynamic, new advances in modeling diseases *in vitro* may help unraveling what is still unknown about primary and secondary events.

Whilst animal studies have clearly been (and still are) valuable tools in understanding (patho)physiological signaling pathways underlying heart and kidney communication, we have argued here that innovative *in vitro* models will soon contribute to a deeper understanding and modeling of CRS in humans. For this, the convergence of human stem cell biology with engineering technologies will enable control of environmental cues, the development of tailored assays and the design of relevant read-outs that reflect what occurs in patients. Cardiorenal OoC technology has the potential to be the next “best-in-class” platform to precisely model human CRS, allowing drug screening and development of novel treatments.

## Author Contributions

All authors outlined, drafted, wrote, and approved the final version of the manuscript.

## Conflict of Interest

The authors declare that the research was conducted in the absence of any commercial or financial relationships that could be construed as a potential conflict of interest.

## Publisher’s Note

All claims expressed in this article are solely those of the authors and do not necessarily represent those of their affiliated organizations, or those of the publisher, the editors and the reviewers. Any product that may be evaluated in this article, or claim that may be made by its manufacturer, is not guaranteed or endorsed by the publisher.

## Abbreviations

ACM: arrhythmogenic cardiomyopathy
LV: left ventricular
ADMA: asymmetric dimethylarginine
LVH: left ventricular hypertrophy
AGE: advanced glycation endproduct
MCP-1: monocyte chemoattractant protein-1
AKI: acute kidney injury
MI: myocardial infarction
ANP: atrial natriuretic peptides
MMP: matrix metalloproteinase
ATP: adenosine triphosphate
NADPH: nicotinamide adenine dinucleotide phosphate
BNP: B-type natriuretic peptide
NO: nitric oxide
CKD: chronic kidney disease
NOX: NAPDH oxidase
CRS: cardiorenal syndrome
ODN: oligodeoxynucleotide
CV: cardiovascular
OoC: organ-on-a-chip
ECM: extracellular matrix
PGC1 α: proliferator-activated receptor gamma coactivator 1 α
EndoMT: endothelial-to-mesenchymal transition
RAAS: renin-angiotensin-aldosterone system
EMT: epithelial-to-mesenchymal transition
ROS: reactive oxygen species
FGF: fibroblast growth factor
α -SMA: α -Smooth muscle actin
Gal-3: Galectin-3
SNS: sympathetic nervous system
GFR: Glomerular filtration rate
STNx: 5/6 subtotal nephrectomy
HF: heart failure
TGF- β: transforming growth factor- β
hPSC: human pluripotent stem cell
TIMP: tissue inhibitor of metalloproteinase
hiPSC: human induced pluripotent stem cell
TMAO: trimethyl amine N-oxide
ICAM: intercellular adhesion molecule
TNF- α: tumor necrosis factor- α
IL: interleukin
UUO: unilateral ureteral obstruction.
